# Machine learning for predicting diabetes risk in western China adults

**DOI:** 10.1186/s13098-023-01112-y

**Published:** 2023-07-27

**Authors:** Lin Li, Yinlin Cheng, Weidong Ji, Mimi Liu, Zhensheng Hu, Yining Yang, Yushan Wang, Yi Zhou

**Affiliations:** 1 Zhongshan School of Medicine, Sun Yat-sen University, No. 74, Zhongshan Second Road, Yuexiu District, Guangzhou, 510080 Guangdong China; 2 People’s Hospital of Xinjiang Uygur Autonomous Region, No. 91 Tianchi Road, Tianshan District, Urumqi, 830001 Xijiang China; 3 Center of Health Management, The First Affiliated Hospital of Xinjiang Medical University, No. 393, Xinyi Road, Xinshi District, Urumqi, 830054 Xinjiang China

**Keywords:** Diabetes mellitus, Machine learning, Risk prediction model, XGBoost, physical examination

## Abstract

**Objective:**

Diabetes mellitus is a global epidemic disease. Long-time exposure of patients to hyperglycemia can lead to various type of chronic tissue damage. Early diagnosis of and screening for diabetes are crucial to population health.

**Methods:**

We collected the national physical examination data in Xinjiang, China, in 2020 (a total of more than 4 million people). Three types of physical examination indices were analyzed: questionnaire, routine physical examination and laboratory values. Integrated learning, deep learning and logistic regression methods were used to establish a risk model for type-2 diabetes mellitus. In addition, to improve the convenience and flexibility of the model, a diabetes risk score card was established based on logistic regression to assess the risk of the population.

**Results:**

An XGBoost-based risk prediction model outperformed the other five risk assessment algorithms. The AUC of the model was 0.9122. Based on the feature importance ranking map, we found that hypertension, fasting blood glucose, age, coronary heart disease, ethnicity, parental diabetes mellitus, triglycerides, waist circumference, total cholesterol, and body mass index were the most important features of the risk prediction model for type-2 diabetes.

**Conclusions:**

This study established a diabetes risk assessment model based on multiple ethnicities, a large sample and many indices, and classified the diabetes risk of the population, thus providing a new forecast tool for the screening of patients and providing information on diabetes prevention for healthy populations.

**Supplementary Information:**

The online version contains supplementary material available at 10.1186/s13098-023-01112-y.

## Introduction

Diabetes mellitus (DM) is a metabolic disease characterized by hyperglycemia. Hyperglycemia can cause chronic damage to tissues over time [1]. Diabetes has become a major health problem worldwide with a significant increase in DM patients. According to the International Diabetes Federation (IDF), approximately 537 million adults worldwide had diabetes in 2021 (with a prevalence of 10.5%), and it is estimated that by 2045, approximately 783 million people worldwide are likely to have diabetes (with a prevalence of approximately 12.2%) [2, 3]. In China, the number of adults with diabetes ranked first in the world in 2021 (approximately 140.9 million patients, with a prevalence rate of approximately 13.0%) [3, 4]. According to a survey, because individuals with type-2 diabetes mellitus (T2DM) usually lack the relevant knowledge, or they are asymptomatic, some individuals with T2DM patients can not be detected in time (approximately 50% of individuals with T2DM are undiagnosed) [3, 5]. It is necessary to identify individuals with diabetes in the population in an efficient and accurate manner. Therefore that early preventive measures and treatment can be taken to avoid further escalation of T2DM.

Currently, the scientific community has shifted its focus to the use of powerful computational methods for early and accurate prediction of diabetes [6–11]. Machine learning (ML) can iteratively learn nonlinear interactions from large amounts of data [12–14]. At present, based on electronic medical records and hospitalization data, ML methods have been used in the diagnosis and prediction of diabetes, prediabetes, complications and disease progression [7, 8, 15–17], as well as real-time blood glucose monitoring [18, 19], with some success. However, most of these models are created for the care of T2DM patients, and the sample size of training data is too small to reliably capture asymptomatic cases of early abnormal blood glucose, which are not suitable for mass screening of the population or public health planning [20, 21]. One study [22] reported that most models for diabetes prediction and risk assessment were rarely used because they relied on specific data. As physical examination data grows and ML rapidly develops, the use of physical examination data for disease risk assessment can provide better clinical guidance and facilitate large-scale screenings at an earlier stage [23]. However, at present, fewer scholars conduct diabetes screening based on health examination data [8, 24]. ML methods have not been applied to T2DM screening models and risk assessment in western China based on large-scale physical examination data.

We aimed to develop an ML model suitable for large-scale screening of T2DM among adults in western China. In this study, we established the model based on logistic regression (LR) and ML algorithms, including classification and regression tree (CART), light gradient boosting machine (LightGBM), random forest (RF), extreme gradient boosting (XGBoost), multilayer perceptron (MLP), and TabNet model, and combined them with western China large-scale health examination data, which are characterized by wide coverage, large volume and strong representation. In addition, in order to improve the convenience and flexibility of the model, a diabetes risk score card was established based on logistic regression to assess the risk of the population. This study is the first T2DM screening model that systematically compares various algorithms on a multiethnic and large sample basis.

## Materials and methods

### The dataset

We used the health examination data obtained from the national physical examination (NPE) project in 2020, which was previously described in detail [25]. The NPE health examination consisted of three parts: questionnaire, routine physical examination and laboratory tests.

**Fig. 1:**
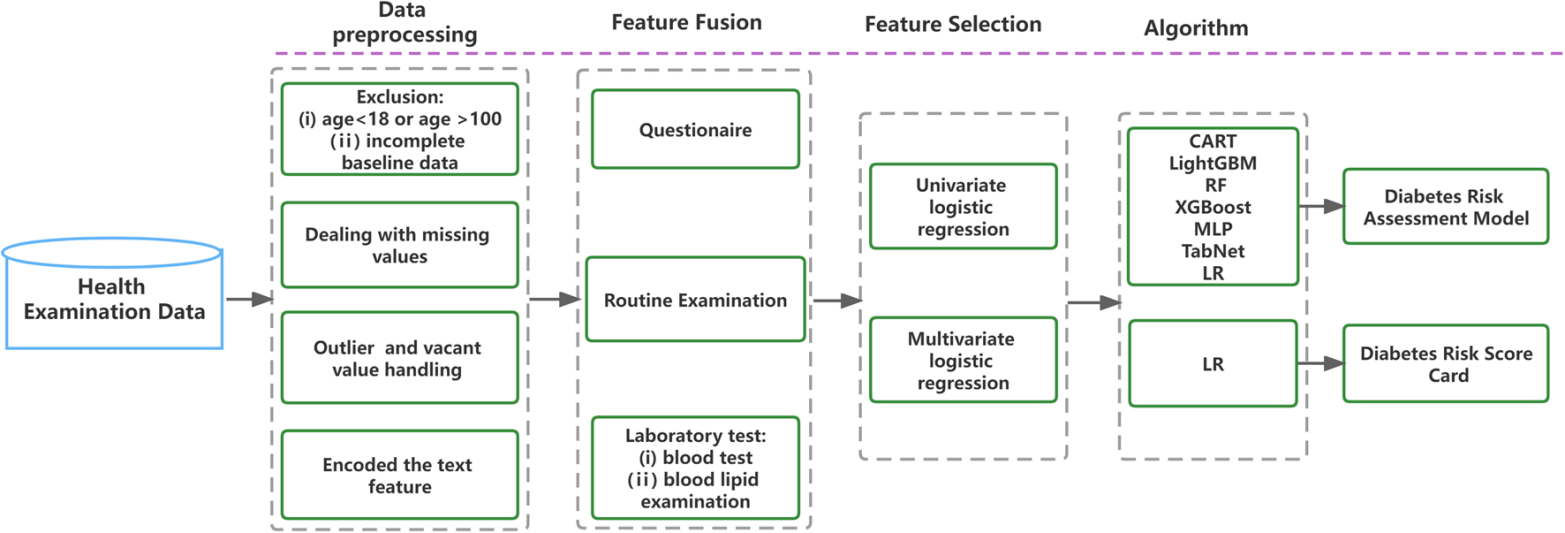
Flow Chart. *CART* classification and regression tree, *LightGBM* light gradient boosting machine, *RF* random forest, *XGBoost* extreme gradient boosting, *LR* logistic regression

A total of 9,333,091 people were enrolled in this study by signing an informed consent form. Participants were excluded from the study if they were (i) younger than 18 years old; or (ii) more than 20% of their baseline and laboratory test data were missing. Second, we removed variables unrelated to the study, such as participants’ names, contact phone numbers, and home addresses. After that, missing value processing (random forest interpolation) and extreme value processing (deletion) were performed for the remaining variables. The detailed analysis process is shown in Fig. 1. Finally, 4,075,431 samples were left, including 3,774,084 healthy individuals and 3,013,47 T2DM patients.

### Feature fusion

In our computational model, we combined three types of physical examination data: questionnaire data (9 features), routine tests (2 features), and laboratory values (9 features). A total of 20 features were sufficient to identify diabetes risk. Through the questionnaire, we collected demographic characteristics, diet, smoking, hypertension, coronary heart disease, and parental history of T2DM in the population. Body mass index (BMI) and waist circumference (WC) were collected through routine tests. Through laboratory testing, nine laboratory values were collected.

T2DM was defined if any of the following criteria were met: 2 h postprandial blood glucose (2hPG) ≥ 11.1 mmol/L, fasting blood glucose (FBG) ≥ 7.0 mmol/L, or a complaint of diabetes and the use of antidiabetic drugs.

### Feature selection

To adjust the parameters and measure the model’s performance, the data were segmented using the 70–30 holdout method. The training set contained 2852801 samples (healthy population: 2641683, T2DM patients 211118). The possible risk factors for DM were preliminarily screened by reviewing the relevant literature. Univariate and multivariate logistic regression analyses were performed to analyze these characteristics, and correlation analysis was used to determine the correlation between each characteristic.

### Classification algorithms

In this study, integrated learning (CART, LightGBM, RF, XGBoost), deep learning (TabNet and MLP) and LR models were used to construct a diabetes risk assessment model.

The CART algorithm is a tree arrangement algorithm. CART has the advantages of fast operation speed, high accuracy, high-dimensional data and no parameter assumptions. There are some problems with it, including high variance and overfitting, which limit its applicability as an independent prediction model.

The RF algorithm is a combination of bagging ensemble learning theory and the random subspace method [26, 27]. The core idea of the RF algorithm is to construct multiple independent classifiers, and then apply the average or majority voting principle to their predictions to determine the results of ensemble classifiers.

The XGBoost technique is a nonlinear machine learning technique based on trees [8]. XGBoost is based on combining weak estimators to predict hard-to-evaluate samples repeatedly [28], so as to constitute a strong estimator. The XGBoost can evaluate the importance of each input feature more easily than other black box techniques such as support vector machine (SVM) and artificial neural network (ANN) techniques.

The LightGBM algorithm is a decision tree-based ensemble algorithm that provides an effective implementation of gradient lifting [29]. Compared to traditional training algorithms, LightGBM has a faster training speed, a lower memory requirement, and a higher accuracy, which can lead to more efficient models.

MLP is a feed-forward, supervised artificial neural network structure that can contain multiple hidden layers through multilayer perceptrons to achieve classification modeling of nonlinear data.

TabNet is a neural network for tabular data that uses sequential attention mechanism to select the features to be reasoned about at each decision step, thus learning to obtain the most salient features for interpretability and more efficient learning.

In order to facilitate clinical and real-life applications, we designed a diabetes risk scorecard based on LR. In the process of establishing the score card, we used the chi-square method for continuous variables, and the discrete variables were directly divided into categories. We determined the final number of boxes according to the information value (IV) value curve. Then, the IV value of each feature was calculated and variables whose IV value was greater than 0.1 were selected into the scorecard model. Finally, the weight of evidence (WOE) value of each box was calculated, and the WOE was mapped back to the original dataset, and then LR was used to establish the model. The detailed process can be found in a previous study [8].

### Model evaluation

To obtain the optimal parameters, we used grid search to perform hyperparameter debugging on four models to obtain the optimal parameters. Based on the confusion matrix, we calculated the accuracy, recall, sensitivity, specificity, positive predictive value (PPV), negative predictive value (NPV), and receiver operating characteristic (ROC) curve of each model. Furthermore, we utilized the Kolmogorov–Smirnov (KS) value to appraise the efficiency of the scorecard model. A higher value of KS is indicative of an improved model. The greater the KS value, the more successful the model is. The KS value, which varies from 0 to 1, and when KS surpasses 0.3, the prediction performance of the model is deemed satisfactory.

### Statistical analysis

The baseline characteristics of the study population are represented as the mean ± standard deviation when they are continuous variables, and as frequency (percentage) when they are categorical variables.

The differences in variables between diabetic patients with diabetes and healthy people were analyzed. The t test or Mann–Whitney test was used for continuous variables. The chi-square test or Fisher’s exact test were used for categorical variables. Statistical significance was inferred at a two-sided P-value < 0.05.

This study utilized Python Software Version 3.8.3. The libraries “Pandas” “NumPy” and “Matplotlib” were used for determining nulls and outliers as well as for interpolation. Meanwhile, the “Sklearn” library was used for construction and validation of the ML model. We use “PyTouch” to build a deep learning framework.

## Results

### Basic characteristics

A total of 9,333,091 participants were included in this study. After data preprocessing, 4,075,431 participants were left, including 1,919,248 (47.09%) males and 2,156,183 (52.91%) females. A total of 3,774,084 healthy people and 301,347 T2DM patients were included. The prevalence of T2DM was calculated at 7.39% among the study population. The general characteristics of the study population are presented in Table 1. Patients with diabetes had older age, higher BMI, WC, HGB, WBC, FB, TC, TG, LDLC, lower PLT and HDLC than healthy people. Compared with the healthy population, the proportion of patients with hypertension and CAD was higher in diabetic patients with diabetes. The prevalence of T2DM was significantly different among people with different dietary habits and smoking statuses. For further details, see Table 1.

**Table 1:** Characteristics of participants in this study

Category	Features	Health (n = 3774084)	Diabetes (n = 301347)	p value
Questionnaire	Sex, n(%)			< 0.001
	Male	1778128 (47.11%)	141120 (46.83%)	
	Female	1995956 (52.89%)	160227 (53.17%)	
	Age(year)	49.33 ± 15.58	61.77 ± 11.13	< 0.001
	Ethnicity, n(%)			< 0.001
	Uyghur	2015933 (53.42%)	133687 (44.36%)	
	Han	1106565 (29.32%)	128512 (42.65%)	
	Kazak	369059 (9.78%)	12591 (4.18%)	
	Hui	177841 (4.71%)	21256 (7.05%)	
	Khalkha	36773 (0.97%)	1025 (0.34%)	
	Mongol	34752 (0.92%)	1479 (0.49%)	
	Tajik	6655 (0.70%)	105 (0.03%)	
	Other	26506 (0.18%)	2692 (0.89%)	
	EH, n(%)			< 0.001
	Balanced diet	3647207 (96.64%)	289846 (96.18%)	
	Meat based	54428 (1.44%)	4466 (1.48%)	
	Vegetarian based	72449 (1.92%)	7035 (2.33%)	
	SS,n(%)			< 0.001
	Never smoked	3352894 (88.84%)	271177 (89.99%)	
	Smoking	396921 (10.52%)	26578 (8.82%)	
	Quit smoking	24269 (0.64%)	3592 (1.19%)	
	HTN,n(%)			< 0.001
	No	2977467 (78.89%)	109105 (36.21%)	
	Yes	796617 (21.11%)	192242 (63.79%)	
	CAD,n(%)			< 0.001
	No	3609085 (95.63%)	252633 (83.83%)	
	Yes	164999 (4.37%)	48714 (16.17%)	
	PDM,n(%)			< 0.001
	No	3742806 (99.17%)	294209 (97.63%)	
	Yes	31278 (0.83%)	7138 (2.37%)	
Routine examination	WC (cm)	86.36 ± 11.46	91.39 ± 11.46	< 0.001
	BMI (kg/m2)	25.08 ± 3.89	26.65 ± 3.76	< 0.001
Laboratory test	HGB, g/L	140.96 ± 16.65	143.11 ± 15.5	< 0.001
	The WBC, × 109/L	6.29 ± 1.47	6.6 ± 1.5	< 0.001
	PLT, × 109/L	235.08 ± 57.5	227.88 ± 57.92	< 0.001
	FBG, mmol/L	5.19 ± 0.69	5.81 ± 0.68	< 0.001
	ECG,n(%)	0.21 ± 0.41	0.3 ± 0.46	< 0.001
	Normal	2982273 (79.02%)	212044 (70.37%)	
	Abnormal	791811 (20.98%)	89303 (29.63%)	
	TC, mmol/L	4.41 ± 0.96	4.68 ± 1.01	< 0.001
	TG, mmol/L	1.26 ± 0.54	1.49 ± 0.56	< 0.001
	LDLC, mmol/L	2.48 ± 0.8	2.62 ± 0.86	< 0.001
	HDLC, mmol/L	1.36 ± 0.36	1.32 ± 0.36	< 0.001

For continuous variables, the data are expressed as the mean ± standard deviation, and for categorical variables, the data are expressed as counts (percentage)

*DM* diabetes mellitus, *EF* exercise frequency, *EH* eating habits, *SS* smoking status, *HTN* hypertension, *CAD* coronary heart disease, *PHTN* parental hypertension, *PDM* parental diabetes mellitus, *PCHD* parental coronary heart disease, *MS* marital status, *WC* waist circumference, BMI body mass index, *SBP* systolic blood pressure, *DBP* diastolic blood pressure, *HGB* hemoglobin, *WBC* white blood cell, *PLT* platelet, FBG fasting blood glucose, *ECG* electrocardiogram, *TC* total cholesterol, *TG* triglyceride, *LDLC* low-density lipoprotein cholesterol, *HDLC* high-density lipoprotein cholesterol

**Fig. 2:**
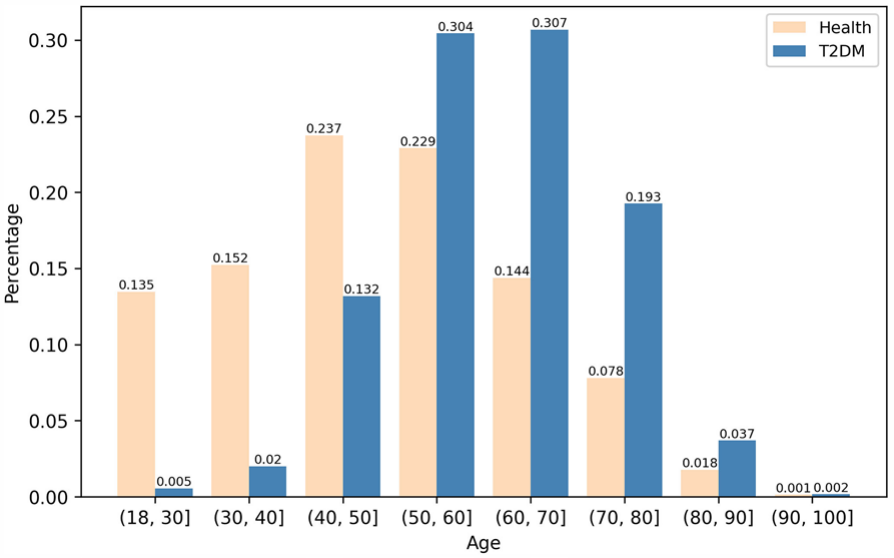
Distribution of diabetes patients and healthy people by age. Healthy people (yellow) and T2DM patients (blue)

We compared the prevalence of diabetes in different age groups (Fig. 2). It was found that the age of diabetes patients was concentrated in the range of 50–80 years old, accounting for approximately 60% of diabetes patients. Diabetes patients younger than the age of 40 accounted for 2.5% of the total number of diabetes patients.

### Feature selection

The possible risk factors for T2DM were preliminarily screened by reviewing relevant literature (Table 2). The Pearson’s Correlation Coefficient was utilized to reveal the interrelationship between the various features. The correlation between the factors was then depicted using heat maps (Additional file 1: Figure A1). In Additional file 1: Figure A1, BMI had a positive correlation with WC, while HTN and CDA showed a positive correlation.

**Table 2:** Multivariate logistic regression analysis in the development group

Category	Features	Multivariate logistic regression analysis		
		Beta	OR(95%CI)	P value
Questionnaire	Sex, n(%)			
	Male			
	Female	0.1095	1.116 (1.103 1.129)	< 0.001
	Age(year)	0.0299	1.03 (1.030 1.031)	< 0.001
	Ethnicity, n(%)			
	Uyghur			
	Han	0.384	1.469 (1.452 1.485)	< 0.001
	Kazak	0.549	0.578 (0.564 0.592)	< 0.001
	Hui	0.465	1.592 (1.56 1.624)	< 0.001
	Khalkha	0.732	0.481 (0.445 0.52)	< 0.001
	Mongol	0.524	0.592 (0.554 0.632)	< 0.001
	Tajik	1.09	0.335 (0.264 0.419)	< 0.001
	Other	0.142	1.153 (1.094 1.215)	< 0.001
	EH, n(%)			
	Balanced diet			
	Meat based	0.0762	1.079 (1.037 1.123)	< 0.001
	Vegetarian based	0.018	1.018 (0.985 1.052)	0.282349
	SS, n(%)			
	Never smoked			
	smoking	0.0645	1.067 (1.048 1.086)	< 0.001
	Quit smoking	0.171	1.186 (1.131 1.244)	< 0.001
	HTN, n(%)			
	No			
	Yes	1.212	3.361 (3.324 3.398)	< 0.001
	CAD, n(%)			
	No			
	Yes	0.471	1.603 (1.579 1.627)	< 0.001
	PDM, n(%)			
	No			
	Yes	1.310	3.706 (3.575 3.841)	< 0.001
Routine examination	WC (cm)	0.0121	1.012 (1.012 1.013)	< 0.001
	BMI (kg/m2)	0.0147	1.015 (1.013 1.017)	< 0.001
Laboratory test	HGB, g/L	0.001	0.999 (0.999 0.999)	< 0.001
	WBC, × 10^9^/L	0.126	1.134 (1.13 1.138)	< 0.001
	PLT, × 10^9^/L	0.002	0.998 (0.998 0.998)	< 0.001
	FBG, mmol/L	0.946	2.574 (2.556 2.593)	< 0.001
	ECG, n(%)			
	TC, mmol/L	0.005	1.005 (0.999 1.012)	0.090
	TG, mmol/L	0.255	1.29 (1.278 1.303)	< 0.001
	LDLC, mmol/L	0.019	0.981 (0.974 0.988)	< 0.001
	HDLC, mmol/L	0.196	0.822 (0.810 0.833)	< 0.001

*EH* eating habits, *SS* smoking status, *HTN* hypertension, *CAD* coronary heart disease, *PDM* parental diabetes mellitus, *WC* waist circumference, *BMI* body mass index, *HGB* hemoglobin, *WBC* white blood cell, *PLT* platelet, *FBG* fasting blood glucose, *ECG* electrocardiogram, *TC* total cholesterol, *TG* triglyceride, *LDLC* low-density lipoprotein cholesterol, *HDLC* high-density lipoprotein cholesterol

Univariate logistic regression analysis (Additional file 1: Table A1) and a multivariate logistic regression analysis (Table 2) were performed for these features. We found that age, unbalanced diet, smoking, hypertension, CAD, PDM, WC, BMI, WBC, FGB, TC, and TG were positively associated with the risk of T2DM. HDL was negatively associated with T2DM. Multivariate logistic regression showed that HGB, PLT and LDLC were negatively correlated with the risk of T2DM, which may be related to the data itself and affected by missing values. Considering that some previous studies found a relationship between TC and T2DM, combined with correlation analysis and logistic regression analysis, finally, sex, age, ethnicity, EH, SS, HTN, CAD, PDM, WC, BMI, WBC, PLT, FBG, ECG, TC, TG, LDLC, and HDLC were chosen to construct the diabetes risk prediction model.

### Tuning of the parameters

To obtain the optimal parameters, we used grid search and cross-validation to conduct hyperparameter debugging for seven models, as shown in Additional file 1: Table A2.

### Comparison of model performance

In this study, we constructed various tree-based machine learning models, such as CART, LightGBM, RF, XGBoost, MLP and TabNet, as well as the LR model. Table 3 and Additional file 1: Figure A2 show the performance of each prediction model on the validation group. The results showed that XGBoost had a good model performance, with an AUC of 0.9122. XGBoost also showed superiority in accuracy (0.8314), precision (0.2800), PPV (0.9829) and NPV (0.9122). Table 3 demonstrates the efficacy of each prediction model on the validation group.

**Table 3:** Performance metrics of the machine learning models

Models	Accuracy	Sensitivity	Specificity	PPV	NPV	AUC
CART	0.7870	0.8181	0.7845	0.2322	0.9819	0.8839
LightGBM	0.7799	0.8237	0.7764	0.2269	0.9822	0.8808
RF	0.7663	0.8217	0.7619	0.2156	0.9817	0.8730
XGBoost	0.8314	0.8180	0.8324	0.2800	0.9829	0.9122
MLP	0.8008	0.7803	0.8025	0.2394	0.9787	0.8754
TabNet	0.8068	0.7728	0.8095	0.2443	0.9781	0.8759
LR	0.9260	0.07522	0.9938	0.4918	0.93097	0.8161

*PPV* positive predictive value, *NPV* negative predictive value, *AUC* area under the receiver operating characteristic curve

**Fig. 3:**
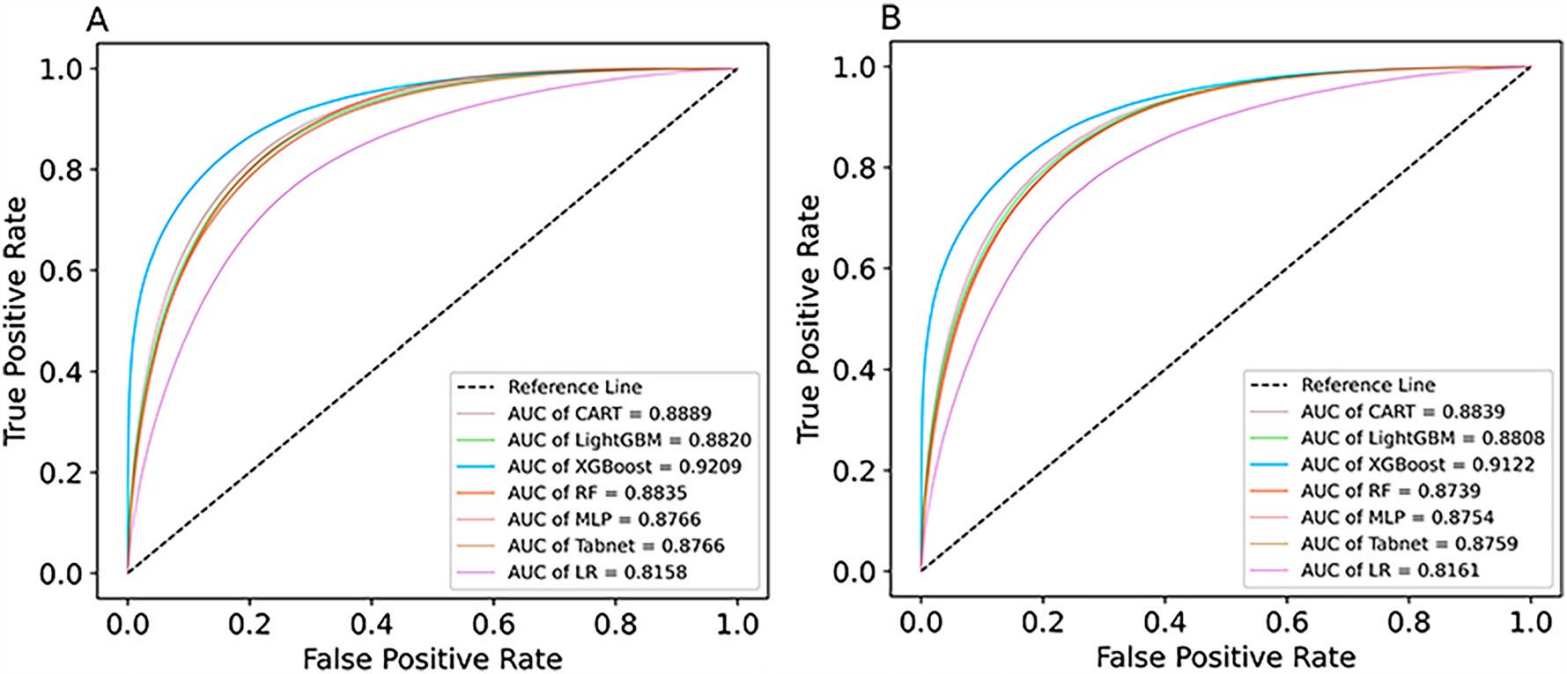
ROC curves of different learning machine learning algorithms on the training and validation sets. **A** ROC curve in the development group. **B** ROC curve in the validation group

Figure 3 shows the ROC curves and AUC of different prediction models in the development group and validation group. It is found that XGBoost performed better than the other prediction models. The AUC of the development group was 0.9209, and the AUC of the validation group was 0.9122. The results showed that the XGBoost algorithm showed excellent advantages in predicting the risk of diabetes in this study.

**Fig. 4:**
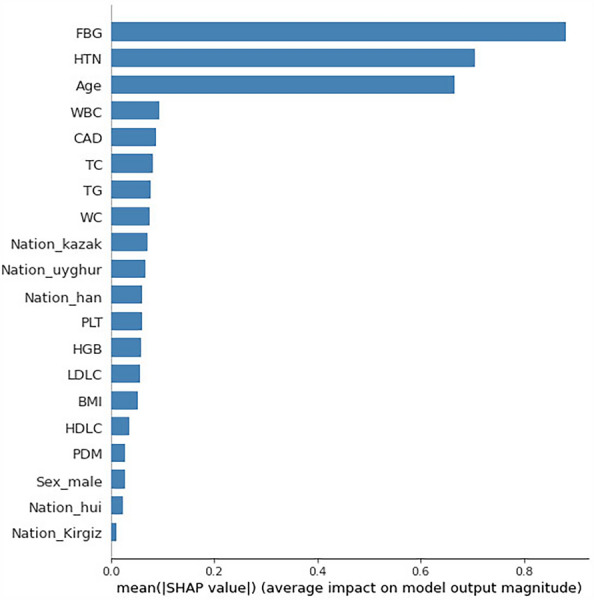
Feature importance of the XGBoost model. *HTN* hypertension, *FBG* fasting blood glucose, *PDM* parental diabetes mellitus, *CAD* coronary heart disease, *WC* waist circumference, *BMI* body mass index, *WBC* white blood cell, *HGB* hemoglobin, *PLT* platelet, *TC* total cholesterol, *TG* triglyceride, *LDLC* low density lipoprotein cholesterol, *HDLC* high density lipoprotein cholesterol

We used SHapley Additive Explanations (SHAP) to explain the characteristic contributions of the XGBoost model. Figure 4 showes the feature importance of the XGBoost algorithms. We found that HTN, FGB, age, PDM, CAD, ethnicity, TG, WC, BMI and TC were identified as the top ten of the most important factors.

### Diabete risk score card

**Fig. 5:**
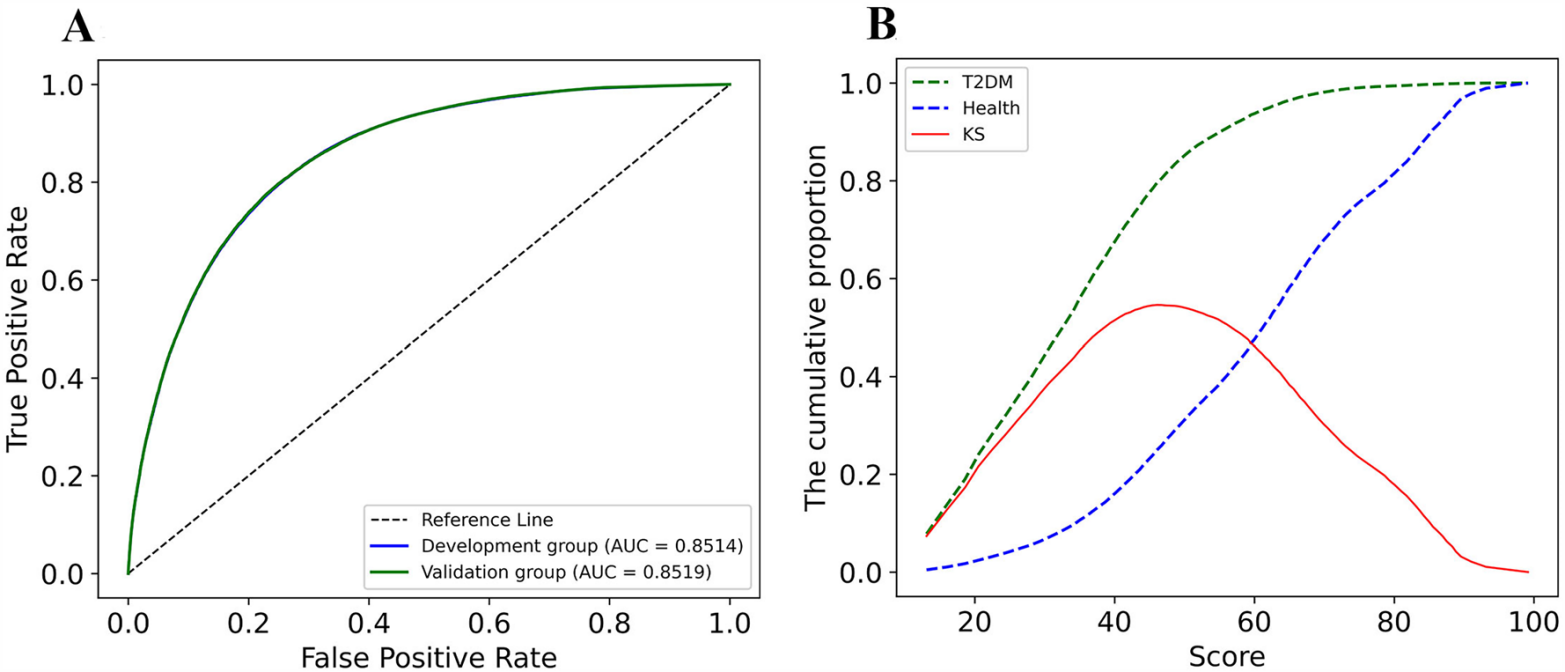
ROC and KS curves of the diabetes risk score card. **A** ROC curve; **B** KS curve

A diabetes risk score card with a scale of 100 was designed for this study. The diabetes risk score card was used to evaluates an individual's risk of diabetes by aiding in the calculation of their risk score. Based on the IV value, the risk score card model was established using age, FBG, HTN, WC, BMI, TG, CAD and ethnicity as variables. The ROC and KS curves of the validation group are displayed in Fig. 5.

We used the score card scaling algorithm to convert the model into score cards (Table 4). The score card comprises the baseline score as well as the associated score for each box within each feature. When using a scorecard, the total score is the sum of the base score and the feature score, which represents the diabetes risk value. In this study, the base score was 46.3.

**Table 4:** Diabetes risk score card

Feature	Threshold	Score	Feature	Threshold	Score
Age	(-inf, 39.0]	17.75	CDA	No	0.60
	(39.0, 46.0]	6.39		Yes	− 5.97
	(46.0, 50.0]	2.86	Ethnicity	Uyghur	1.65
	(50.0, 56.0]	− 1.02		Han	− 3.32
	(56.0, inf]	− 5.28		Kazak	7.52
FGB	(-inf, 5.3]	11.04		Hui	− 3.58
	(5.3, 5.6]	1.69		Khalkha	9.33
	(5.6, 5.8]	− 4.56		Mongol	5.54
	(5.8, 6.1]	− 9.43		Other	− 0.51
	(6.1, 6.43]	− 15.07	WC	(-inf, 82.0]	2.13
	(6.43, inf]	− 11.60		(82.0, 90.0]	0.17
BMI	(-inf, 20.4]	1.87		(90.0, 102.0]	− 1.23
	(20.4, 22.86]	1.08		(102.0, inf]	− 2.55
	(22.86, 24.28]	0.37	TG	(-inf, 0.62]	3.84
	(24.28, 26.48]	− 0.20		(0.62, 1.01]	1.95
	(26.48, inf]	− 0.74		(1.01, 1.34]	0.69
HTN	No	6.26		(1.34, 1.6]	− 0.62
	Yes	− 8.90		(1.6, inf]	− 2.41

The Kolmogorov–Smirnov curve (Fig. 5) was utilized to illustrate the totality of the score and to determine the risk interval. The higher the KS value is, the greater the segmentation ability of the model’s corresponding threshold value will be. As illustrated in Fig. 5, the apex of the inflection point is achieved when the score is equal to 45. Therefore, to easily calculate the risk interval, we set 50 as the intermediate threshold. The higher the score generated from testing, the lower the risk of diabetes; conversely, the lower the score, the greater the likelihood of developing diabetes. To supply users with a more direct evaluation, four risk categories have been established in accordance with the KS chart (Table 5).

**Table 5:** Risk interval division and threshold of diabetes risk score card

Score	Proportion of health	Proportion of diabetes	Risk group
0–25	4.16%	33.10%	Very high
25–50	26.94%	52.02%	High
50–75	44.53%	13.93%	Normal
75–100	24.37%	0.95%	low

### Comparison with existing models

To further validate the efficacy of our model, a comparison of the proposed model against other leading methods was conducted, the results of which are presented in Table 6.

**Table 6:** Comparison with existing models

Author	Feature	Method	AUC
Gao et al. [30]	Age, Sex, WC, Systolic pressure and PDM	LR	0.635
Yang et al. [8]	BMI, FGB, Waist-toheight ratio, Age, Mean systolic pressure, Urine glucose	XGBoost	0.881
Zhou et al. [31]	Age, Sex, Systolic pressure, BMI, WC, PDM	LR	0.748
Ravaut et al. [16]	demographics, routine diagnosis codes and history, laboratory values, geographical information prescription history, information on the specialty of each doctor encounter, and hospitalizations	XGBoost	80.26
This study	Sex, Age, Ethnicity, EH, SS, HTN, CAD, PDM, WC, BMI, WBC, PLT, FBG, ECG, TC, TG, LDLC, HDLC	XGBoost	0.9122

## Discussion

The increasing burden of diabetes has become a global challenge [3, 32]. Through mass screening, early identification and intervention of patients with diabetes can be achieved to delay or prevent the development of the disease [33, 34]. The most efficacious method for widespread screening of diabetes has yet to be identified. In this study, the T2DM risk prediction models were developed and validated on data from more than 4 million people. The data were obtained from the cross-sectional data of NPE, including more than 9 million people in 14 prefectures of Xinjiang, China, which can be considered representative of the overall population of Xinjiang. Following the evaluation of the model's performance, it was determined that the XGBoost model was the optimal model for predicting the risk of T2DM, with an AUC was is 0.9122.

In this study, we used questionnaires to obtain indicators of hypertension and cardiovascular diseases, genetic history and smoking and diet in the population, which not only captured the medical history of each patient, but also included demographic factors and laboratory test indicators. Univariate and multivariate logistic regression analyses showed that sex, age, ethnicity, EH, SS, HTN, CAD, PDM, WC, BMI, WBC, PLT, FBG, ECG, TC, TG, LDLC, and HDLC were important factors for diabetes. HTN, FGB, age, PDM, CAD, ethnicity, TG, WC, BMI, and TC were the most important predictors of diabetes. Except that the FGB was viewed as a recognized risk factor and predictor of T2DM, hypertension and CAD were the most important features of T2DM risk models, which presented with high predictive ability. Some studies have confirmed that hypertension, cardiovascular disease and diabetes are mutually promote and influence each other [35, 36]. Many pathophysiological mechanisms underlie the association between diabetes and cardiovascular disease. Among these mechanisms, several have been identified as potential contributors [36]. Including insulin resistance in the nitric-oxide pathway, the stimulatory effect of hyperinsulinemia on sympathetic drive, smooth muscle growth, and sodium-fluid retention, as well as the excitatory effect of hyperglycemia on the renin–angiotensin–aldosterone system, provide plausible explanations for the association between diabetes and cardiovascular disease. On the other hand, the functional changes occurring in the context of T2DM and hypertension significantly alter the hemodynamic stress on the heart and other organs. Some studies have also demonstrated the important role of ECG in the prediction of diabetes [37], and our study confirmed the association between abnormal ECG results and T2DM. Understanding these underlying mechanisms is crucial for developing targeted interventions to prevent and manage cardiovascular complications in individuals with diabetes.

Our study showed that age was also an important feature of diabetes prediction models. The FDRSMA is a classic and widely used diabetes risk scoring model [38]. The objective of FDRSM is to utilize six risk factors (including BMI, FBG, PDM, HDLC, blood pressure and TG) to evaluate the risk of T2DM among middle-aged individuals. T2DM is generally observed in adults and appears to be more prevalent among the elderly individuals. As people age, the glucose sensitivity of pancreatic cells decline and insulin secretion is impaired, leading to hyperglycemia and T2DM [39]. Several studies reported differences in the incidence of diabetes between ethnic groups [40–42] and confirmed that ethnicity could be a predictors of diabetes [40, 43–46]. In our study, we used Uyghur as a reference, with Han and Hui ethnic groups exhibiting a heightened susceptibility to diabetes. The kazakh, Mongolian and Tajik ethnic groups had a lower risk. Genetic and environmental differences (i.e., economic level, diet, lifestyle, climate) were taken into account. Family history of diabetes was also identified as an important risk factor for T2DM in our model, which is consistent with previous studies [47]. There is a significant genetic predisposition to T2DM, with a 2 to 30 fold increased risk for T2DM in those with a family history compared with those without a family history [48].

Many studies have demonstrated a connection between obesity and diabetes. Furthermore, our study discovered that augmented BMI and WC were correlated with a higher probability of having diabetes. The development of obesity gain can result in insulin resistance and diminished β-cell functionality in humans. According to the World Health Organization, the global increase in the prevalence of diabetes is believed to be related to chronic stress, being overweight [49], lacking of physical activity [50, 51], excessive consumption of alcohol [52, 53] and an unhealthy diet [54]. Our model also demonstrated that EH and SS were predictors of T2DM. In addition, we also found that people who smoked and those who had quit smoking had a higher risk of T2DM than those who did not smoke, and those who ate a vegetarian or meat-based diet had a higher probability of T2DM than those who ate a balanced meat-vegetarian diet.

We incorporated laboratory variables, including TC, TG and HDLC into the diabetes prediction model. Our findings indicated that TG was an independent risk factor for T2DM, while TC was not an independent risk factor for T2DM in our study. Consistent with other studies [55]. The feature importance ranking showed that TC, TG, LDLC and HDLC were all important features of the T2DM risk prediction model. Multiple studies have revealed that dyslipidemia and T2DM often coexist in individuals and share common pathological mechanisms, such as insulin resistance, metabolic disturbances, inflammation, and alterations in the gut microbiota [55, 56].

Currently, ML algorithms are increasingly used to predict diabetes and related diseases [11, 12, 18, 19, 30, 57–59]. In this study, a diabetes screening model based on CART, LightGBM, RF, XGBoost TabNet and MLP models was constructed. The AUC (0.9122), PPV (0.2800), NPV (0.9829) and accuracy (0.8314) of the XGBoost prediction model showed good performance in the validation group. It appears that our model outperforms the majority of existing models, which may be because the model is built on the basis of multiple features and big data. Other studies also found that XGBoost was effective in predicting the risk of diabetes [8, 16].

The development of the diabetes risk assessment score card assists clinicians and individuals alike in conducting self-examinations, with the aim of increasing the rate of diabetes cascade screening and enhancing individual lifestyle management. Hence, utilizing large-scale physical examination information to achieve prompt risk notification and identification of diabetes is the most practicable course of action.

This study has several advantages. First, based on the NPE project, it not only has a wide coverage and a large amount of data, but also includes a number of major ethnic groups in China, which can enable better assessment of the characteristics of the population in Xinjiang, China; in addition the risk prediction model has a good generalization ability in Xinjiang, China. Second, the risk factors affecting diabetes were fully considered in this study. Laboratory examination, questionnaire survey and routine examination data were fully taken into account to obtain indicators such as hypertension and cardiovascular diseases, genetic history and exercise and diet in the population, and the influencing factors of diabetes were comprehensively analyzed. Third, the results of our model all showed satisfactory predictive effects (XGBoost: AUC = 0.9122). This study also has several limitations. First, it is not possible to establish causality using cross-sectional data derived from national health examinations, therefore, these results should be subject to further investigated in subsequent research. Second, the health examination data used in our study were highly heterogeneous and had a high rate of missing data, which affected the power of the model.

## Conclusion

T2DM imposes an inexorable and significant burden on society, including intangible costs of lost productivity, premature death, and poor quality of life. Our model is based on large-scale health examination data in Xinjiang, China, which was used to construct a large-scale early diabetes risk screening model. Our model can be applied directly to the physical examination database, providing a highly efficient means for the identification of high-risk diabetes records over at a large range. This allows for the understanding of potential diabetes risk ratios at the public health level and the implementation of more effective diabetes prevention and control strategies. It is of great significance for the early control of diabetes to identify early risk warning sings and perform screening based on large-scale physical examination data.

## Supplementary Information

**Additional file 1: Table A1.** Univariate logistic regression analysis. **Table A2.** Hyperparameters of the model. **Figure A1.** The heat map to show the Pearson correlation of features. **Figure A2.** Confusion matrix of each classification model. (A) CART, (B) LightGBM, (C) RF, (D) XGBoost, (E) MLP, (F) TabNet and (G) LR.

## Data Availability

The data used to support the findings of this study are available from the corresponding author upon request.
