# Supplementary material for: Machine learning for predicting diabetes risk in western China adults
Source: Diabetol Metab Syndr. 2023 Jul 27;15:165. doi: 10.1186/s13098-023-01112-y (PMC10373320; doi:10.1186/s13098-023-01112-y)
Supplement: Supplementary file 1 — Additional file 1: Table A1. Univariate logistic regression analysis. Table A2. Hyperparameters of the model. Figure A1. The heat map to show the Pearson correlation of features. Figure A2. Confusion matrix of each classification model. (A) CART, (B) LightGBM, (C) RF, (D) XGBoost, (E) MLP, (F) TabNet and (G) LR. [file 13098_2023_1112_MOESM1_ESM.docx]

**Appendix A**

**Table A1.** Univariate logistic regression analysis

| **Category** | **Features** | **β** | **OR(95%CI)** | **ROC(95%CI)** |
| --- | --- | --- | --- | --- |
| Demographics | Sex,n(%) |  |  |  |
|  | male |  |  |  |
|  | female | 0.0117 | 1.012(1.003-1.021) | 0.501(0.5-0.503) |
|  | age(year) | 0.0553885 | 1.057(1.057-1.057) | 0.739(0.737-0.74) |
|  | Nation,n(%) |  |  | 0.593(0.591-0.595) |
|  | Uyghur | Reference |  |  |
|  | Han | 0.560 | 1.751(1.734-1.768) |  |
|  | Kazak | -0.662 | 0.516(0.504-0.527) |  |
|  | Hui | 0.590 | 1.804(1.771-1.837) |  |
|  | Khalkhas | -0.866 | 0.421(0.39-0.453) |  |
|  | Mongol | -0.439 | 0.644(0.605-0.686) |  |
|  | Tajik | -1.374 | 0.253(0.201-0.314) |  |
|  | Other | 0.426 | 1.532(1.46-1.606) |  |
| Questionnaire | EH,n(%) |  |  | 0.502(0.502-0.503) |
|  | Balanced diet |  |  |  |
|  | Meat based | 0.0397 | 1.041(1.003-1.079) |  |
|  | Vegetarian based | 0.196 | 1.217(1.181-1.254) |  |
|  | SS,n(%) |  |  | 0.512(0.511-0.513) |
|  | Never |  |  |  |
|  | Smoking | -0.182 | 0.833(0.821-0.846) |  |
|  | Quit smoking | 0.588 | 1.801(1.726-1.878) |  |
|  | HTN,n(%) |  |  |  |
|  | No |  |  |  |
|  | Yes | 1.883 | 6.574(6.513-6.636) | 0.714(0.712-0.716) |
|  | CAD,n(%) |  |  |  |
|  | No |  |  |  |
|  | Yes | 1.438 | 4.215(4.16-4.27) | 0.559(0.558-0.56) |
|  | PDM,n(%) |  |  |  |
|  | No |  |  |  |
|  | Yes | 1.071 | 2.918(2.828-3.009) | 0.508(0.507-0.508) |
| Routine examination | WC (cm) | 0.0378 | 1.039(1.038-1.039) | 0.625(0.623-0.627) |
|  | BMI (kg/m^2^) | 0.099 | 1.104(1.103-1.106) | 0.618(0.616-0.619) |
| Laboratory test | HGB, g/L | 0.0079 | 1.008(1.008-1.008) | 0.538(0.537-0.54) |
|  | WBC,×10^9^/L | 0.138 | 1.148(1.144-1.151) | 0.561(0.559-0.563) |
|  | PLT, ×10^9^/L | -0.002 | 0.998(0.998-0.998) | 0.538(0.536-0.54) |
|  | FBG, mmol/L | 1.253 | 3.501(3.478-3.524) | 0.756(0.754-0.758) |
|  | ECG,n(%) |  |  |  |
|  | TC, mmol/L | 0.286 | 1.331(1.325-1.338) | 0.579(0.577-0.581) |
|  | TG, mmol/L | 0.705 | 2.023(2.008-2.039) | 0.618(0.616-0.62) |
|  | LDLC, mmol/L | 0.212 | 1.236(1.23-1.243) | 0.55(0.548-0.552) |
|  | HDLC, mmol/L | -0.309 | 0.734(0.725-0.743) | 0.53(0.528-0.532) |

**Table A2.** Hyperparameters of the model

| **Model** | **Super parameter** |
| --- | --- |
| CART | bagging_temperature: 1; depth: 1; 2_leaf_reg: 6; learning_rate: 0; N_estimators: 6500.0 |
| LightGBM | boosting_type: gbdt; is_unbalance: True;n_jobs: -1; seed: 3008; Learning_rate: 0.06; min_child_weight: 7; max_depth: 10; Colsample_bytree: 0.60; Subsample: 0.9165210396581378; n_estimators: 125; num_leaves: 80 |
| RF | n_estimators: 800; class_weight: balanced; n_jobs: -1; random_state: 3008; Min_samples_split: 0.0001; Min_samples_leaf: 0.0001; max_depth: 70; max_features: log2 |
| XGBoost | booster: gbtree; Scale_pos_weight: 12.5; use_label_encoder: False; objective: binary:logistic; reg_alpha: 0; reg_lambda: 1; n_jobs: -1; seed: 3008; Learning_rate: 0.21000000000000002; Gamma: 0.13; min_child_weight: 6; max_depth: 5; Colsample_bytree: 0.5; Subsample: 0.9848983760263232; n_estimators: 525 |
| MLP | batchsize: 4096, criterion: CrossEntropyLoss, optimizer: Adam, lr: 0.001.  MLP((input_layer): Linear(in_features=18, out_features=64, bias=True)  (fc_layers): ModuleList(  (0): Linear(in_features=64, out_features=128, bias=True)  (1): Linear(in_features=128, out_features=256, bias=True)  (2): Linear(in_features=256, out_features=512, bias=True)  (3): Linear(in_features=512, out_features=256, bias=True)  (4): Linear(in_features=256, out_features=128, bias=True)  (5): Linear(in_features=128, out_features=64, bias=True))  (out_layer): Linear(in_features=64, out_features=2, bias=True)  (LogSoftmax): LogSoftmax(dim=1)) |
| Tabnet | Batchsize: 1024, ClassificationSMOTE (p=0.2), optimizer:Adam, lr:0.02.  TabNetClassifier(n_d=8, n_a=8, n_steps=3, gamma=1.3, cat_idxs=[0, 2, 5, 6, 15, 16, 17], cat_dims=[2, 8, 3, 3, 2, 2, 2], cat_emb_dim=2, n_independent=2, n_shared=2, epsilon=1e-15, momentum=0.02, lambda_sparse=0.001, seed=0, clip_value=1, verbose=1, optimizer_fn=<class 'torch.optim.adam.Adam'>, optimizer_params={'lr': 0.02}, scheduler_fn=<class 'torch.optim.lr_scheduler.StepLR'>, scheduler_params={'step_size': 50, 'gamma': 0.9}, mask_type='entmax', input_dim=18, output_dim=2, device_name='auto', n_shared_decoder=1, n_indep_decoder=1) |
| LR | random_state: 3008, penalty: l2, C: 3.91, max_iter: 196 |

CART: Classification And Regression Tree; LightGBM: Light Gradient Boosting Machine; RF: Random Forest, XGBoost: Extreme Gradient Boosting; LR:Logistic Regression

#
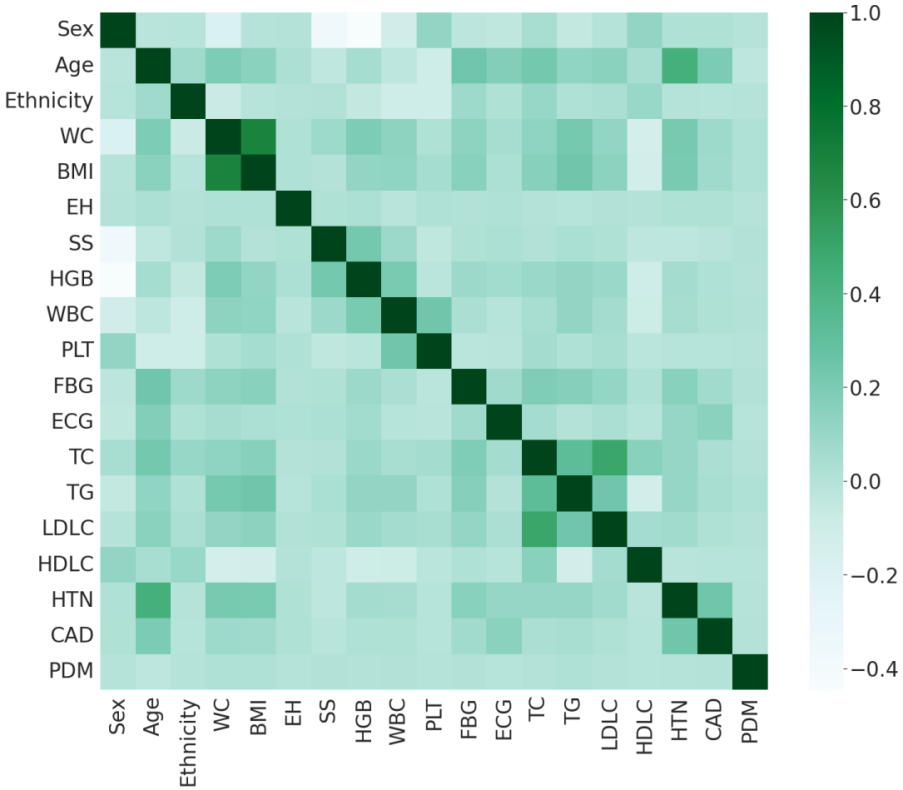


**Figure A1.** The heat map to show the Pearson correlation of features.


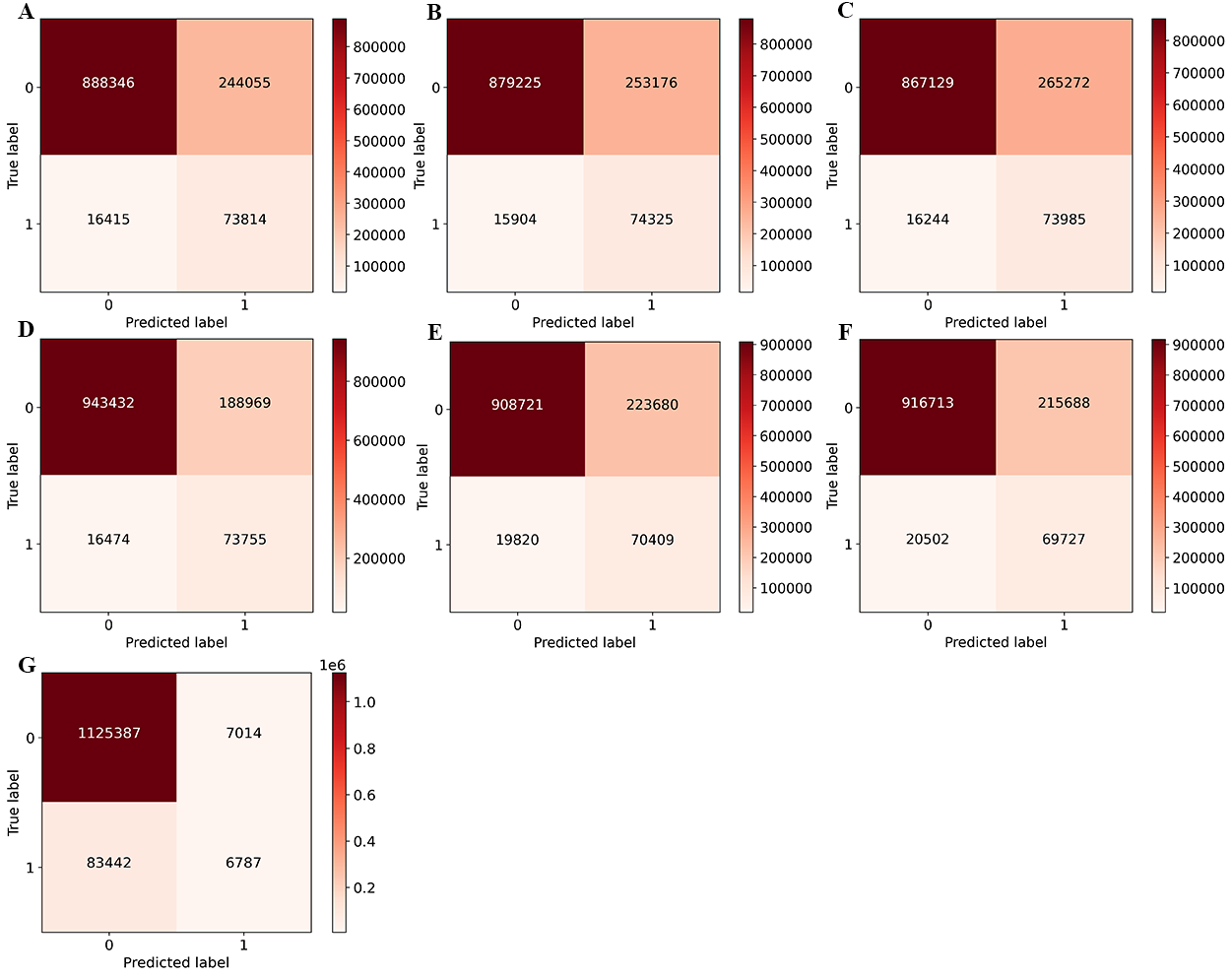


**Figure A2.** Confusion matrix of each classification model. (A) CART, (B) LightGBM, (C) RF, (D) XGBoost, (E) MLP, (F) TabNet and (G) LR.
